# Evaluation of Preferential Cytokine Adsorption onto Biosensing Surface Modified with Glycopolymer

**DOI:** 10.3390/bios15030178

**Published:** 2025-03-12

**Authors:** Yuhei Terada, Masayuki Futamata, Kaori Tsutsui, Hiroshi Aoki

**Affiliations:** Environmental Management Research Institute (EMRI), National Institute of Advanced Industrial Science and Technology (AIST), Tsukuba 305-8569, Japan; y.terada@aist.go.jp (Y.T.);

**Keywords:** glycopolymer, polymer-modified surface, cytokine, molecular recognition, surface plasmon resonance imaging, glycan

## Abstract

For the improvement of biosensor performance, the development of a molecular recognition material as well as a sensor platform is necessary. A glycopolymer is a molecular recognition material capable of recognizing specific proteins as natural glycans. However, the target molecules for biosensors using glycopolymers are limited to lectins that are already known for their specific interactions with glycan residues. The aim of this study is to investigate a glycopolymer-modified (GM) surface capable of recognizing non-lectin proteins. As non-lectin proteins, we focused on cytokines, in which the interaction preference to glycopolymers is unknown. The cytokine adsorption onto the GM surfaces was evaluated using a surface plasmon resonance imaging technique as a biosensing tool. Differences in cytokine adsorption onto the different glycan residues were revealed, which will be important for selective cytokine detection. This study indicates the possibility of a biosensing surface modified with glycopolymers for the detection of non-lectin proteins. The results are beneficial for expanding the use of glycopolymers as a molecular recognition material for future applications such as cell analysis and diagnostic devices.

## 1. Introduction

The demand for biosensors with high sensitivity and selectivity is increasing in fields such as medical engineering and environmental evaluation. Generally, biosensors mainly consist of two parts, the sensor platform (detection part) and the molecular recognition material. Highly sensitive biosensors have been developed by improvements in the sensor platform and sensing techniques, such as electrochemical [1], quartz crystal microbalance [2], and surface plasmon resonance [3]. Molecular recognition materials are also important for improving both the sensitivity and selectivity of biosensors. Generally, biomolecules such as antibodies, nucleic acids, and glycans have been modified onto biosensor surfaces for performing the specific recognition of a target molecule [4]. Such materials were chemically immobilized onto the biosensor surface using alkane thiol [5], silane-coupling reagents [6], or polymer scaffolds [7], which often requires an activation process to form the chemical bond with the molecular recognition material. Time-consuming and multiple processes are required in the method mentioned above, and this is a hindrance to the development of biosensors. Furthermore, biomolecules are fragile when exposed to physical or chemical stimuli such as temperature and pH changes, and delicate handling is also required.

As the molecular recognition material of biosensors, we focus on glycopolymers, artificial polymers presenting a glycan residue on the side chain [8,9,10]. It is capable of recognizing proteins specifically as natural glycans [11], and the interaction with proteins could be enhanced by forming clusters of glycans (cluster glycoside effect) [12,13,14]. In comparison to antibody and natural glycans, glycopolymers are durable against temperature and pH changes due to their polymer-based structure. Chemical modifications with functional groups such as silanes and thiols are possible concurrently with the polymerization process, which leads to direct immobilization of the glycopolymers onto material surfaces [15,16,17]. Furthermore, the drastic development of polymerization techniques in the last few decades enabled the synthesis of glycopolymers with precisely controlled architectures [8,18]. The size and shape of glycopolymers are important for the improvement in versatility, binding capacity, and specificity, which are also critical for developing biosensors with high sensing performance.

Although glycopolymers are attractive as molecular recognition materials, antibodies are widely used in practical biosensors mainly due to variety in the target molecules and their high binding affinity [19,20,21]. Glycopolymers are capable of recognizing proteins specifically, and glycopolymers showing interactions with protein with high affinity have been reported [22,23,24]. However, the target molecules were limited to lectins, which are the proteins already known for interacting with glycans specifically. If a glycopolymer that enables specific or preferential interactions with non-lectin proteins could be found, the use of this glycopolymer for biosensors could be promoted. Herein, we focused on cytokines as the target non-lectin proteins. They are relatively small proteins (<80 kDa) that are especially secreted from immune cells [25,26,27]. Some reports have discussed the recognition of cytokines by glycan [28,29]; however, the details remain unclear. We have previously reported the first detection of cytokine interleukin-2 using a plasmonic biosensor immobilized with mannose-presenting glycopolymer [30], and the potential of cytokine recognition by the glycopolymer was suggested. However, the binding preference among cytokines and glycans remains unknown.

In this research, we investigated the adsorption of cytokines onto a glycopolymer-modified (GM) surface. For the investigation and analysis of such unknown biological interactions, a high-throughput biosensing platform was needed. We adopted the surface plasmon resonance imaging (SPRi) technique as the biosensing platform [31,32]. Biological interactions on the multiple Au spots sputtered onto a glass chip could be measured by monitoring the reflectivity of the light irradiated onto the Au surfaces. The glycopolymers used in this research were synthesized by reversible addition-fragmentation chain transfer (RAFT) polymerization [33,34,35]. This polymerization method enabled the control of polymer length and the presentation of a thiol group at the terminal of the main polymer chain, which enabled the direct immobilization of glycopolymers onto the Au surfaces. The glycopolymers with different types of glycan residue were individually modified on the Au surfaces of the SPRi sensor chip, and the adsorption of cytokines was investigated. We revealed the differences in the adsorption of anti- and pro-inflammatory cytokines onto the GM surfaces. The potential of the GM surface with a specific glycan residue for preferential interaction with the pro-inflammatory cytokine was also indicated. Such differences in the interaction or adsorption properties of cytokines to various GM surfaces possessing different glycan residues were unknown in former research [30]. The preferential adsorption of cytokines to glycans and glycopolymers is beneficial for further understanding glycoengineering. We also expect the use of GM surfaces in bioengineering applications such as cell analysis and diagnostic devices in the future.

## 2. Materials and Methods

### 2.1. Instruments

The key instruments used in this research are as follows: a surface plasmon resonance imaging system, Horizon SPRimager (GWC Technologies, Madison, WI, USA), equipped with a halogen lamp (5 V, 10 W) and a CCD camera (764 × 494 pixels, 800 nm light was detected using a band-pass filter); the analyzing software for SPRi measurement V++ (Ver. 5.0, Digital Optics Ltd., Auckland, New Zealand); a 500 MHz nuclear magnetic resonance (NMR) apparatus JNM-ECA500 (JEOL, Tokyo, Japan); a freeze-drying chamber (FDS-1000, Tokyo Rikakikai Co., Ltd., Tokyo, Japan) equipped with an oil pump (GLD-051, ULVAC KIKO Inc., Miyazaki, Japan); and a Milli-Q grade water system, Milli-Q IQ 7010 (Darmstadt, Germany).

### 2.2. Reagents and Materials

**Figure 1:**
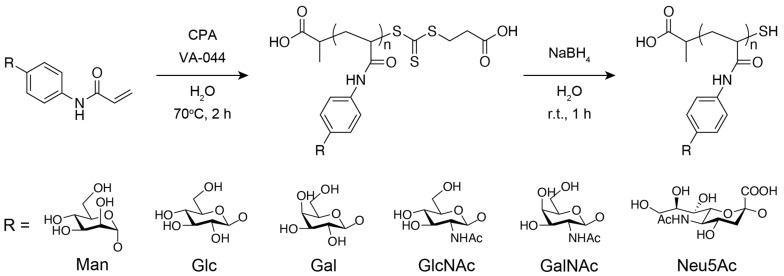
Synthesis of glycopolymers.

The reagents used in this research were purchased from commercial sources and used as received. Monomers with glycan residues, *p*-acrylamidophenyl-α-mannoside (Man), *p*-acrylamidophenyl-β-glucoside (Glc), *p*-acrylamidophenyl-β-galactoside (Gal), *p*-acrylamidophenyl-α-sialoside (Neu5Ac), *p*-acrylamidophenyl-β-*N*-acetylglucosaminide (GlcNAc), and *p*-acrylamidophenyl-β-*N*-acetylgalactosaminide (GalNAc), were purchased from Tokyo Chemical Industry Co., Ltd. (Tokyo, Japan). The structures of each glycan residue are shown in Figure 1. The polymerization initiator 2,2′-azobis[2-(2-imidazolin-2-yl)propane]dihydrochloride (VA-044), sodium borohydride (NaBH_4_), ethanol (EtOH), deuterium oxide (D_2_O), acrylamide, tumor necrosis factor α (TNF-α), interleukin-1α (IL-1α), interleukin-1β (IL-1β), interleukin-6 (IL-6), and PBS(-) (pH 7.2-7.4) were purchased from FUJIFILM Wako Pure Chemical Corp. (Osaka, Japan). A polymerization initiator reagent for the glycopolymer synthesis, 2-(2-carboxyethylsulfanylthiocarbonylsulfanyl)propionic acid (CPA), and a siliconizing reagent, Sigmacote, for the SPRi sensor chip preparation were purchased from Sigma-Aldrich (St. Louis, MO, USA). S-TIH10 glass chips were purchased from ATOCK Co., Ltd. (Tsukuba, Japan).

### 2.3. Preparation of SPRi Sensor Chips

SPRi sensor chips with 28 Au spots with an array interval of 1.6 mm and diameter of 0.8 mm were prepared using a method similar to that of previous studies [36,37,38]. S-TIH10 glass substrates were first rinsed with EtOH and Milli-Q water then dried by N_2_ blow. Sigmacoat was casted onto the glass substrate and incubated for 1 min to form a hydrophobic layer on the surface. The glass substrate was subsequently rinsed with EtOH and Milli-Q water then baked at 100 °C for 5 min. A 30 Å Cr layer was first sputtered onto the hydrophobic glass substrate, and a 450 Å Au layer was sputtered afterwards using a template mask of the SPRi Au spot pattern.

### 2.4. Synthesis of Glycopolymers

The synthesis scheme is shown in Figure 1. Glycopolymers were synthesized using RAFT polymerization, as described in a previous report [30]. Monomers with glycan residues, the RAFT agent CPA, and the initiator VA-044 were dissolved in ~4 mL of Milli-Q water with a 100:1:1 ratio (0.5 M scale) and placed in a Pyrex tube, and the solution was degassed by three freeze–thaw cycles and incubated in a thermostatic oil bath at 70 °C for 2 h under nitrogen atmosphere. Then, 10 μL of the solution was dissolved in 700 μL of D_2_O and used for ^1^H-NMR measurement to determine the monomer conversions to glycopolymers. The rest of the solution was purified by dialysis against the excess amount of Milli-Q water using a dialysis membrane with a molecular weight cutoff of 12,000–14,000 for a day (water was exchanged three times). The purified solution was freeze-dried for over a day. The freeze-dried polymer was dissolved in a NaBH_4_ aqueous solution (NaBH_4_ was added at more than ten times the amount of CPA) and stirred for 1 h to reduce the trithiocarbonate group of the polymer. The solution was purified by dialysis against the excess amount of Milli-Q water using a dialysis membrane with molecular weight cutoff of 12,000–14,000 for a day (water was exchanged three times). The purified solution was freeze-dried again for over a day. As a reference polymer, a 100-mer designed polyacrylamide (PAAm) was synthesized using the same method mentioned above, replacing the glycan monomer with acrylamide, and a dialysis membrane with a molecular weight cutoff of 3500 was used.

### 2.5. General Procedure of SPRi Measurements

**Figure 2:**
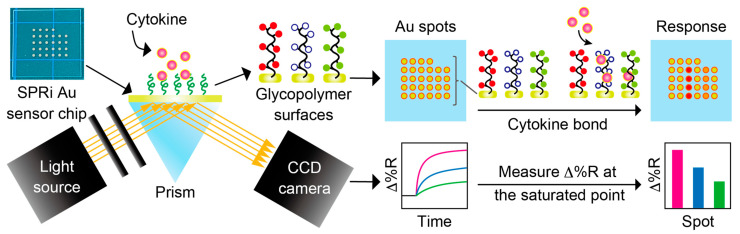
Schematic illustration of SPRi measurement system.

The setup of the SPRi biosensing system is described in Figure 2. In all SPRi measurements, the SPRi chip was rinsed with EtOH and Milli-Q water and dried by nitrogen blow. The chip was placed in a dedicated flow cell, and Milli-Q water or PBS(-) was sent through for over 30 min to remove any unnecessary substances. The reflectivity of the light irradiated onto the chip (Δ%R) automatically calculated by V++ software was monitored during this process to measure the reflectivity baseline, and a stable Δ%R around 0 (±0.3) was determined. Any sample solutions were sent through with a flow rate of approximately 0.1 mL/min, and the solution was circulated during the measurement.

### 2.6. SPRi Measurement of Glycopolymer Immobilization onto SPRi Au Surface

The rinsed SPRi chip with a bare Au spot pattern was set in the flow cell, and the baseline measurement was taken over 30 min in Milli-Q water. Glycopolymer aqueous solutions of 0.31, 0.63, 1.25, 2.50, and 5.0 mg/mL were sent through over 30 min, and the reflectivity change was monitored. The saturation of the reflectivity change at each concentration was confirmed during the measurement, and the Δ%R value increase from the baseline was obtained at the saturated point. Milli-Q water was sent through after the observation to remove physically adsorbed polymers.

### 2.7. SPRi Measurements of Cytokine Adsorption onto GM Surfaces

Glycopolymers were immobilized onto the pre-rinsed SPRi chip by casting 0.5 mL of a 5.0 mg/mL glycopolymer aqueous solution on individual Au spots and incubating them for over 1 h. A glycopolymer concentration of 2.5 mg/mL was necessary for the saturation of glycopolymer immobilization onto the Au surface, as discussed in a later section, and a glycopolymer solution of 5.0 mg/mL was used to ensure the reliable coverage of the Au surface with the glycopolymers. The chip was then sonicated in Milli-Q water and dried by nitrogen blow. The glycopolymer immobilized chip was set in the flow cell, and the baseline measurement was taken over 30 min in PBS(-). A cytokine solution of 200 nM was sent through over 30 min, and the reflectivity change was monitored. The saturation of the reflectivity change at each concentration was confirmed during the measurement, and the Δ%R value increase from the baseline was obtained at the saturated point. PBS(-) was sent through after the observation to remove nonspecifically adsorbed cytokines.

## 3. Results and Discussion

### 3.1. Preparation of Glycopolymer-Modified SPRi Au Surfaces

All glycopolymers synthesized in this research were designed as 100-mer length following the method a previous study [30]. The polymerization of the glycopolymers was analyzed using ^1^H-NMR with D_2_O as the solvent. The conversions of the glycan monomers to the glycopolymers were determined by the ratio of the peaks corresponding to vinyl groups (5.8–6.5 ppm) and the main chain (1.0–2.8 ppm). All glycopolymers were synthesized with conversions of >91%, and it was indicated that the glycopolymers were polymerized successfully. The ^1^H-NMR spectra of the glycopolymers after the purification procedure are summarized in the Supplementary Information.

**Figure 3:**
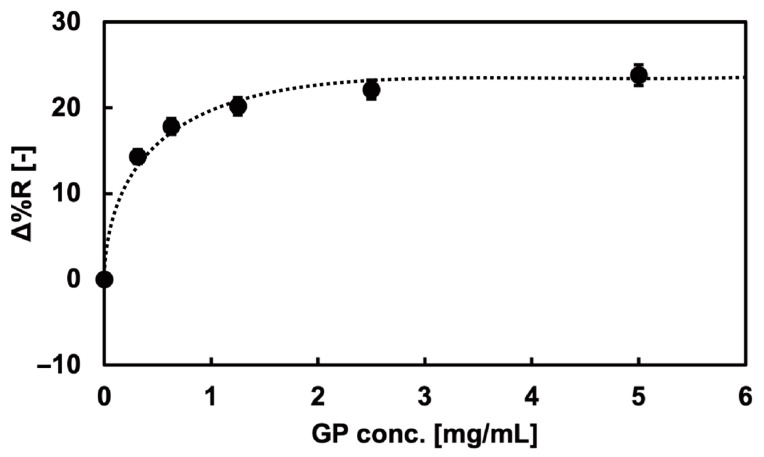
SPRi reflectivity changes obtained by the addition of the glycopolymer solution onto the bare Au surfaces.

Immobilization of the glycopolymers onto the Au surface was measured using the SPRi method. The SPRi reflectivity change (Δ%R) by the 100-mer glycopolymer bond to the Au surface of the SPRi chip is shown in Figure 3. The Δ%R value increased as the concentration of the glycopolymer increased, indicating the immobilization of glycopolymers onto the Au surface. The binding curve showed the Langmuir binding of the glycopolymer, which indicated that the independent bond of each glycopolymer chain and the glycopolymers were uniformly immobilized onto the Au surfaces [39]. Furthermore, the immobilization of the glycopolymer onto the Au surface was saturated at the polymer concentration of 2.5 mg/mL, and it was expected that the Au surface was covered with glycopolymers and that glycan residues were two-dimensionally presented on the Au surface with sufficient density for interaction with the target protein. It was also reported in the previous studies that the area per molecule and the thickness of the 100-mer length glycopolymer was 1.6 nm^2^ [40] and 3.6 nm [39], respectively. The glycopolymer structure used in that report also used a monosaccharide (mannose) as the glycan residue and resembled the glycopolymer used in this research, indicating that the glycopolymer structure formed on the Au surface may be exceedingly close to that of the other report. As a result, it was assumed that a mushroom-like collapsed structured glycopolymer layer was formed on the Au surface [39].

### 3.2. SPRi Measurement of Cytokine Adsorption onto GM Surfaces

**Figure 4:**
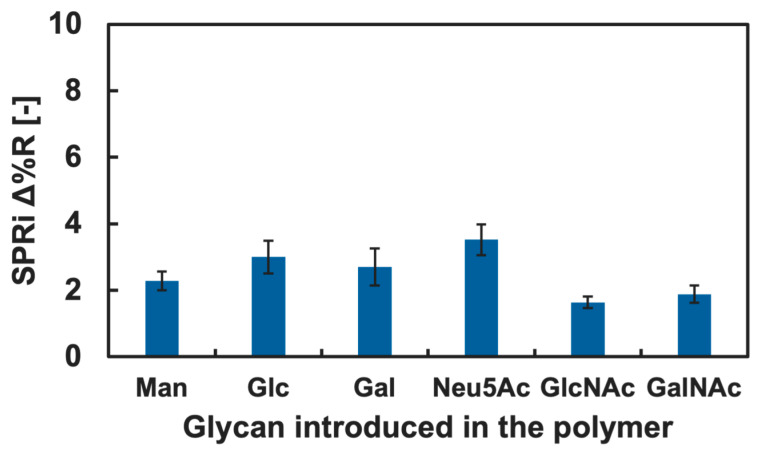
SPRi reflectivity changes obtained by the addition of IL-4 onto the GM surfaces with different types of glycan.

**Figure 5:**
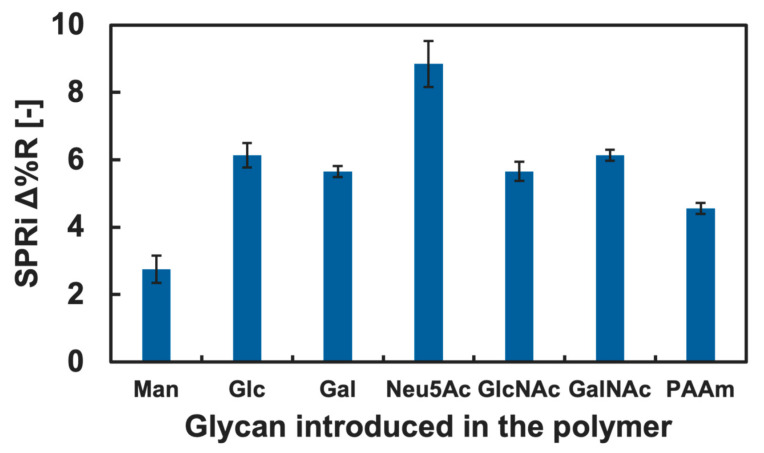
SPRi reflectivity changes obtained by the addition of TNF-α onto the GM surfaces with different types of glycan.

The cytokine adsorption onto the GM surfaces was investigated by measuring changes in Δ%R value when the cytokine solution was added into the SPRi flow cell. Among various cytokines, we firstly focused on IL-4 and TNF-α, known as a type of anti- and pro-inflammatory cytokine, respectively [41,42]. The results of the cytokine adsorption onto the GM surfaces presenting different kinds of glycan residue are shown in Figure 4 and Figure 5. Small changes in the Δ%R value were observed by the addition of the IL-4 solution (Figure 4). The change in the Δ%R value was also observed by the addition of the TNF-α solution, and the amount of change was largest on GM surfaces presenting Neu5Ac residues and was least on the GM surfaces presenting Man residues (Figure 5).

Changes in the Δ%R value by the addition of the blank PBS(-) solution for over 30 min were approximately 0 with deviation of ±0.3, and this was assumed as an instrumentally derived deviation. Considering the amount of change in the Δ%R value (>1.5), we assumed that both IL-4 and TNF-α showed adsorption against the GM surfaces. However, significant differences among the types of glycan residue introduced in the polymers were not observed for the addition of IL-4 (Figure 4), and we assumed that a specific interaction between IL-4 and the GM surfaces was not observed. By contrast, TNF-α indicated slightly preferential adsorption to the GM surfaces presenting Neu5Ac residues compared to the GM surfaces presenting other glycan residues. Furthermore, the changes in the Δ%R value for the polymer surface without glycan residue (PAAm) were smaller than that of the glycopolymer surface with Neu5Ac and even smaller for the GM surfaces with Glc, Gal, GlcNAc, and GalNAc residues (Figure 5). These results indicated that a glycan residue (especially Neu5Ac) was necessary for the interaction with TNF-α.

One possible reason that TNF-α preferred a GM surface presenting Neu5Ac was that the chemical structure differed drastically from other glycan residues. Among the glycan used in the glycopolymers, Neu5Ac was the only glycan with a carboxyl group. It was expected that TNF-α preferred the chemical structure of Neu5Ac or the carboxyl group. Although it is known that the main interaction between protein and glycan is the hydrogen bond, it could be suggested that there was also an electrostatic interaction since the pKa of Neu5Ac is approximately 2.6 [43] and the carboxyl group could be in COO^−^ form in the PBS(-) (pH 7.2–7.4), which may have worked as a cooperative interaction. To confirm the lectin-like binding property, further investigation is needed in a future study. The interactions between cytokines and glycans and glycopolymers are not well known; therefore, the results obtained are expected to be useful for further understanding in glycoengineering and for the development of glycopolymers and glycomaterials capable of the specific recognition of cytokines and other non-lectin proteins.

### 3.3. Evaluation of Cytokine Adsorption Preference onto Glycopolymer Presenting Neu5Ac

**Figure 6:**
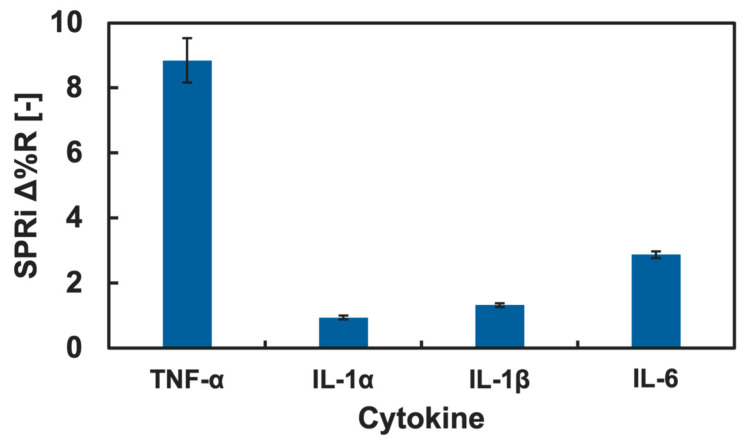
SPRi reflectivity changes obtained by the addition of pro-inflammatory cytokines onto the Neu5Ac-introduced GM surfaces.

TNF-α showed relatively preferential adsorption onto the Neu5Ac-introduced GM surface. However, the binding preference of the Neu5Ac glycopolymers to other cytokines is unknown. We investigated the binding preference of the Neu5Ac-introduced glycopolymer with cytokines using the SPRi technique, and since TNF-α is categorized as a pro-inflammatory cytokine, the adsorption to the Neu5Ac-introduced glycopolymer was compared to other pro-inflammatory cytokines: IL-1α, IL-1β, and IL-6. The SPRi measurement result of pro-inflammatory cytokine adsorption onto the GM surfaces is summarized in Figure 6.

The change in the Δ%R value was largest for TNF-α and was small for IL-1α, IL-1β, and IL-6. The amount of the Δ%R value change was more than three times higher for TNF-α than that of the rest of pro-inflammatory cytokines. This result indicated that a preferential interaction of TNF-α to the GM surface presenting Neu5Ac was observed. The adsorption of IL-1α, IL-1β, and IL-6 to the GM surfaces with Man, Glc, Gal, GlcNAc, and GalNAc was also measured using the SPRi technique, and the results are summarized in the Supplementary Information. The changes in the Δ%R values by the addition of the cytokine (IL-1α, IL-1β, and IL-6) solutions were low for all the GM surfaces, and a significant difference was not observed. Therefore, there were no preferential adsorptions of IL-1α, IL-1β, and IL-6 to any of the GM surfaces. Considering the discussion above, it was indicated that the GM surface presenting Neu5Ac residues had potential for a preferential interaction with TNF-α. Such ability of this GM surface is important for the selective detection of specific cytokines from cell secretion or serum. This GM surfaces is expected for use in selective cytokine detection, which is an important technology for the development in cell analysis and diagnostics.

## 4. Conclusions

The cytokine adsorption to GM surfaces presenting different glycan residues was investigated using the SPRi technique. It was revealed that TNF-α adsorbed onto the GM surfaces compared to the anti-inflammatory cytokine IL-4, and the adsorption was preferential against GM surfaces presenting Neu5Ac residues. The adsorption preference among pro-inflammatory cytokines was also evaluated. Preferential adsorption of TNF-α to the GM surface presenting Neu5Ac was observed and was higher compared to the other pro-inflammatory cytokines (IL-1α, IL-1β, and IL-6). Our research has discovered the potential of GM surfaces for selective cytokine detection, which is an important technology related to cell analysis and diagnostics. Considering that the interaction of cytokines to glycans and glycopolymers is not well known, the results obtained can contribute to further understanding of glycoengineering and the development of glycopolymers with high affinity to non-lectin proteins.

## Data Availability

The datasets generated during and/or analyzed during the current study are available from the corresponding authors upon reasonable request.

## Supplementary Materials

The following supporting information can be downloaded at https://www.mdpi.com/article/10.3390/bios15030178/s1, Figure S1: ^1^H-NMR spectrum of glycopolymer with mannose (Man) residue; Figure S2: ^1^H-NMR spectrum of glycopolymer with glucose (Glc) residue; Figure S3: ^1^H-NMR spectrum of glycopolymer with galactose (Gal) residue; Figure S4: ^1^H-NMR spectrum of glycopolymer with *N*-acetylglucosamine (GlcNAc) residue; Figure S5: ^1^H-NMR spectrum of glycopolymer with *N*-acetylgalactosamine (GalNAc) residue; Figure S6: ^1^H-NMR spectrum of glycopolymer with neuraminic acid (Neu5Ac) residue; Figure S7: ^1^H-NMR spectrum of polyacrylamide; Figure S8: SPRi reflectivity changes obtained by the addition of IL-1α onto the GM surfaces with different types of glycan; Figure S9: SPRi reflectivity changes obtained by the addition of IL-1β onto the GM surfaces with different types of glycan; Figure S10: SPRi reflectivity changes obtained by the addition of IL-6 onto the GM surfaces with different types of glycan.
