# Supplementary material for: Evaluation of Preferential Cytokine Adsorption onto Biosensing Surface Modified with Glycopolymer
Source: Biosensors (Basel). 2025 Mar 12;15(3):178. doi: 10.3390/bios15030178 (PMC11940340; doi:10.3390/bios15030178)
Supplement: Supplementary file 1 [file biosensors-15-00178-s001.zip › biosensors-3468093-supplementary.pdf]

Supplementary Information: Evaluation of Preferential Cytokine Adsorption onto Biosensing Surface modified with Glycopolymer

Yuhei Terada, Masayuki Futamata, Kaori Tsutsui and Hiroshi Aoki *

Environmental Management Research Institute (EMRI), National Institute of Advanced Industrial Science and Technology (AIST), Tsukuba 305-8569, Japan; y.terada@aist.go.jp (Y.T.); futamata.m@aist.go.jp (M.F.); tsutsui.k@aist.go.jp (K.T.)

* Correspondence: aoki-h@aist.go.jp

1. ^{1}H-NMR spectra of 100-mer designed glycopolymers synthesized by RAFT polymerization

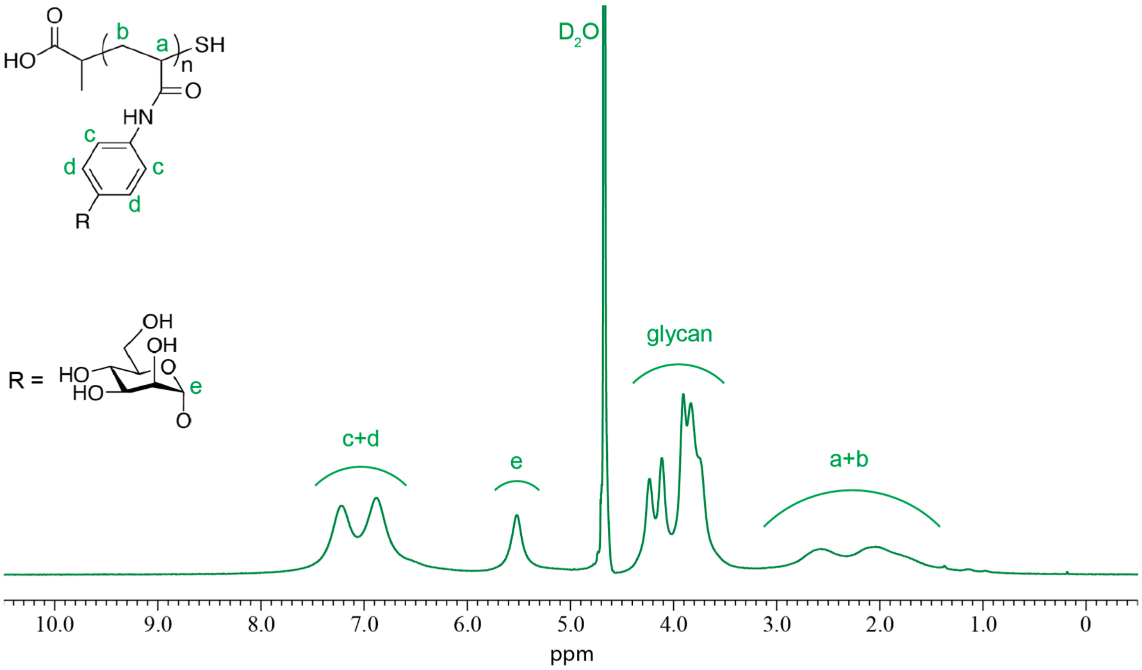

Figure S1. ^{1}H-NMR spectrum of glycopolymer with mannose (Man) residue.

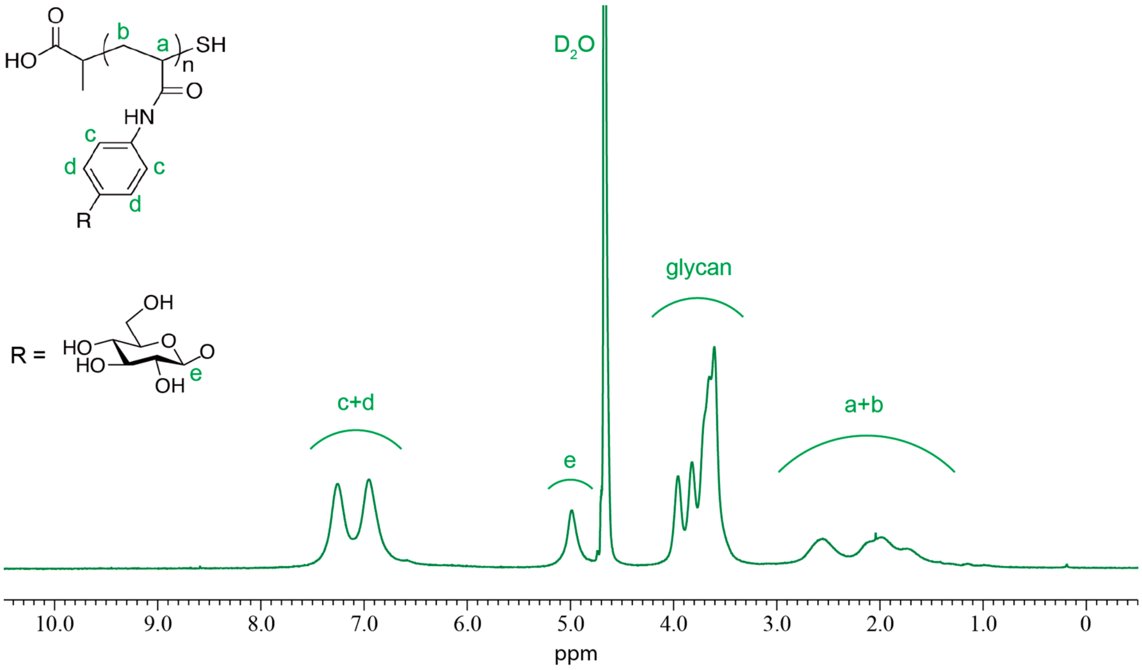

Figure S2. ^{1}H-NMR spectrum of glycopolymer with glucose (Glc) residue.

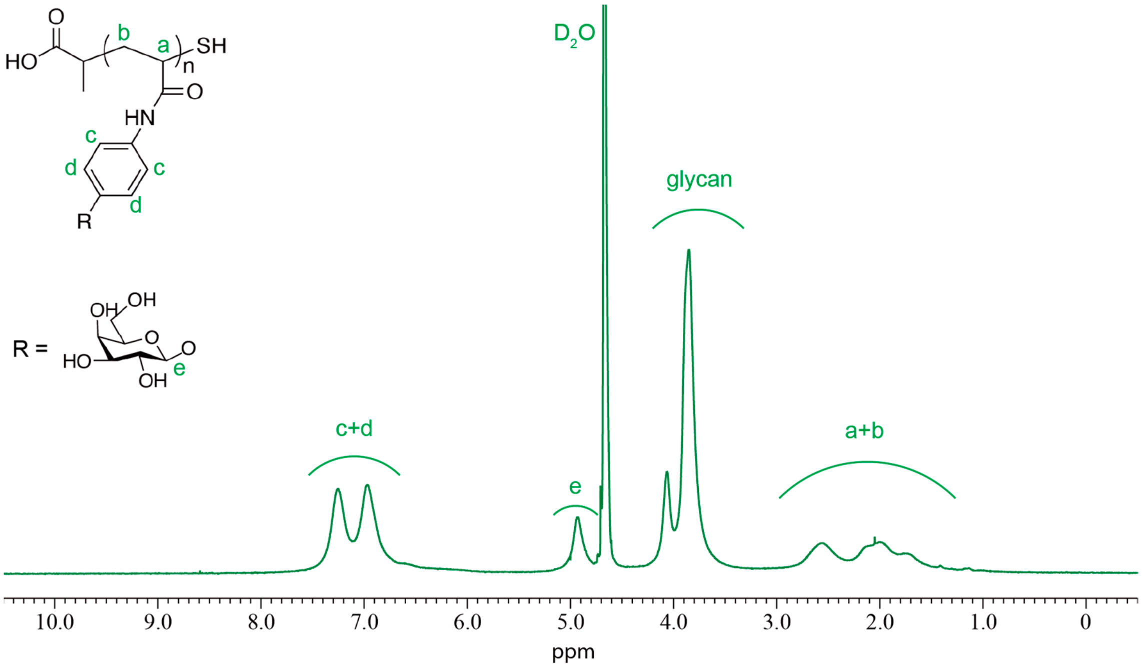

Figure S3. ^{1}H-NMR spectrum of glycopolymer with galactose (Gal) residue.

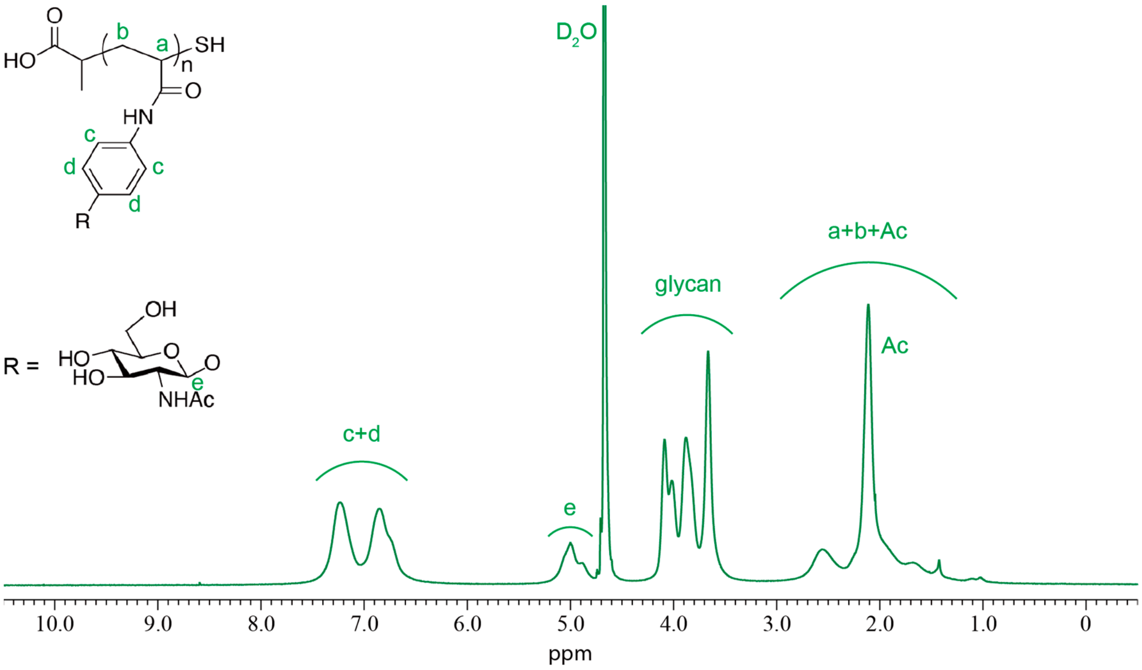

Figure S4. ^{1}H-NMR spectrum of glycopolymer with *N*-acetylglucosamine (GlcNAc) residue.

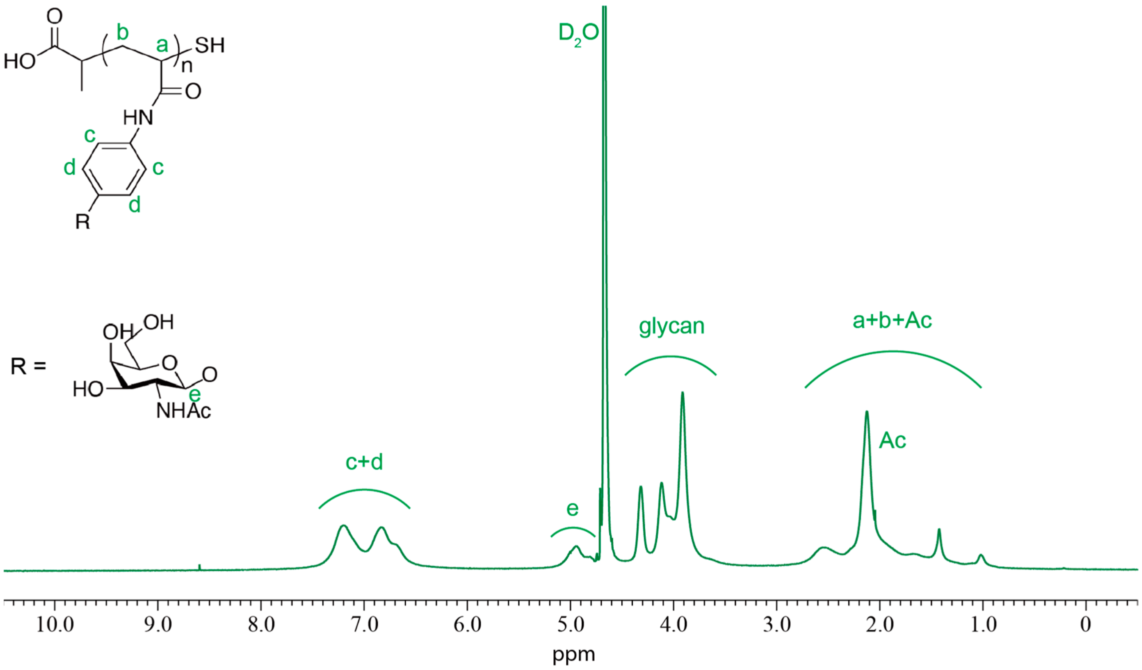

Figure S5. ^{1}H-NMR spectrum of glycopolymer with *N*-acetylgalactosamine (GalNAc) residue.

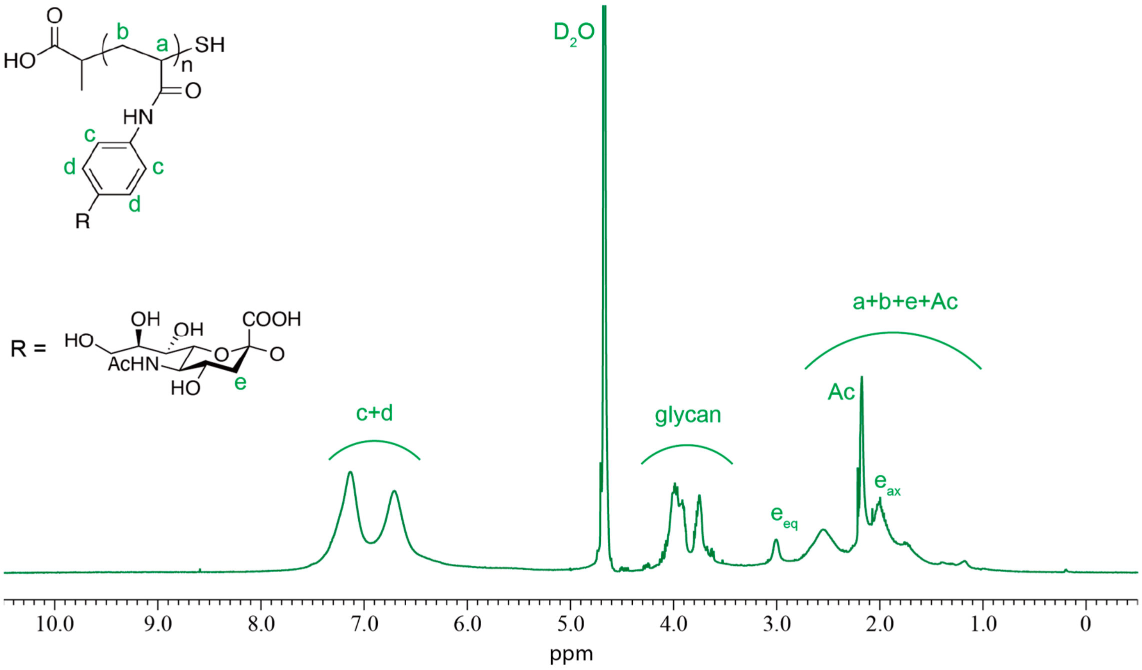

Figure S6. ^{1}H-NMR spectrum of Glycopolymer with neuraminic acid (Neu5Ac) residue.

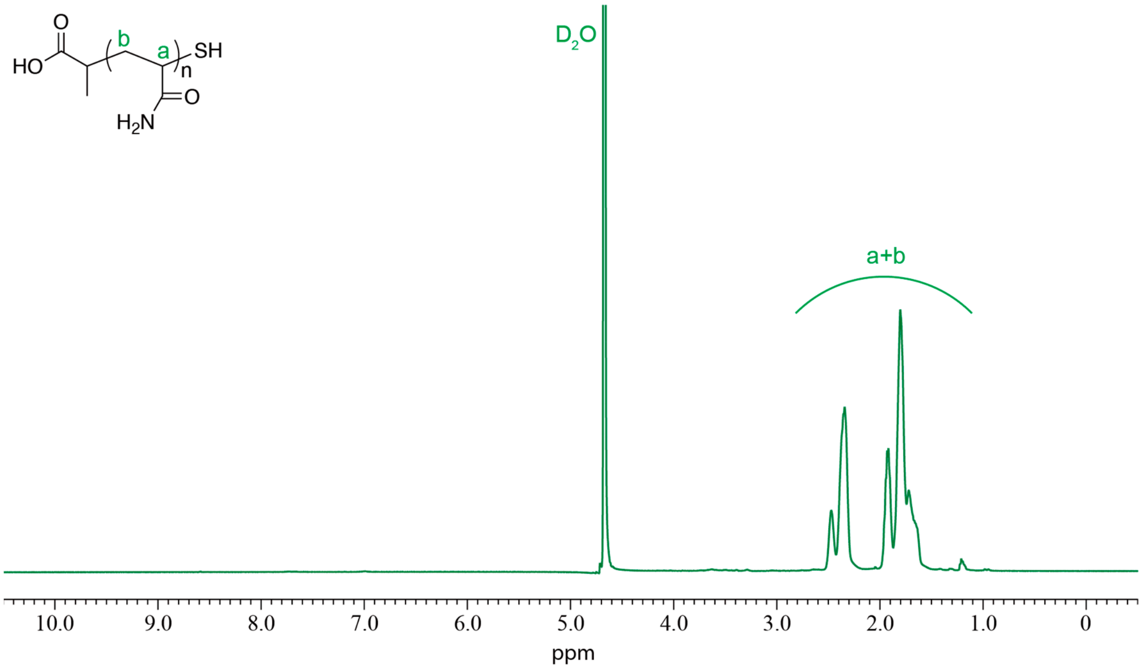

Figure S7. ^{1}H-NMR spectrum of polyacrylamide.

2. SPRi measurement results of cytokine binding onto GM surfaces

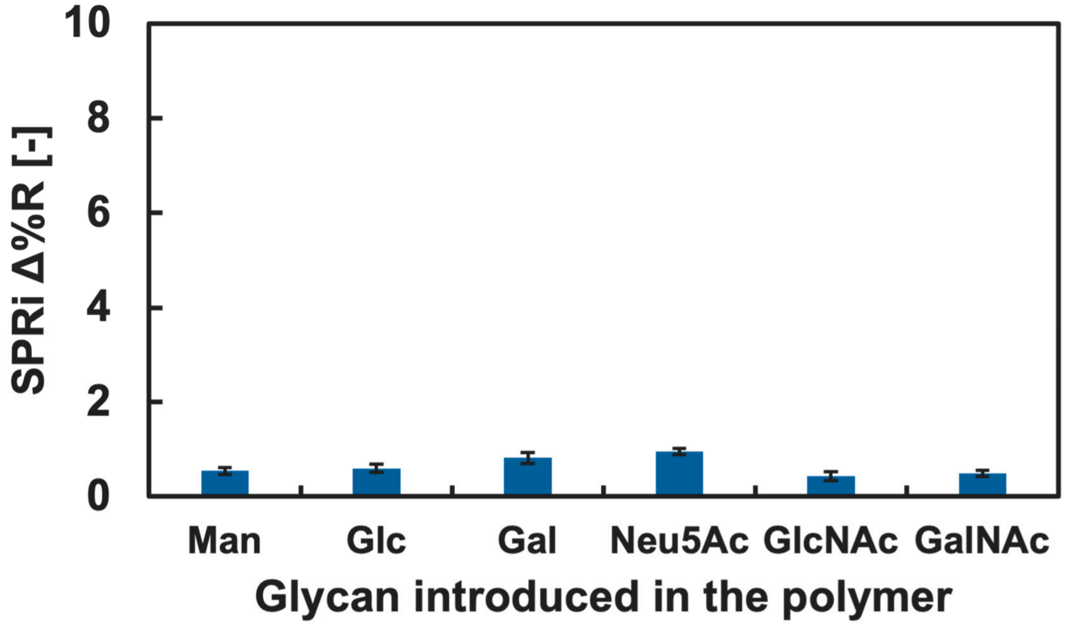

Figure S8. SPRi reflectivity changes obtained by addition of IL-1α onto the GM surfaces with different type of glycan.

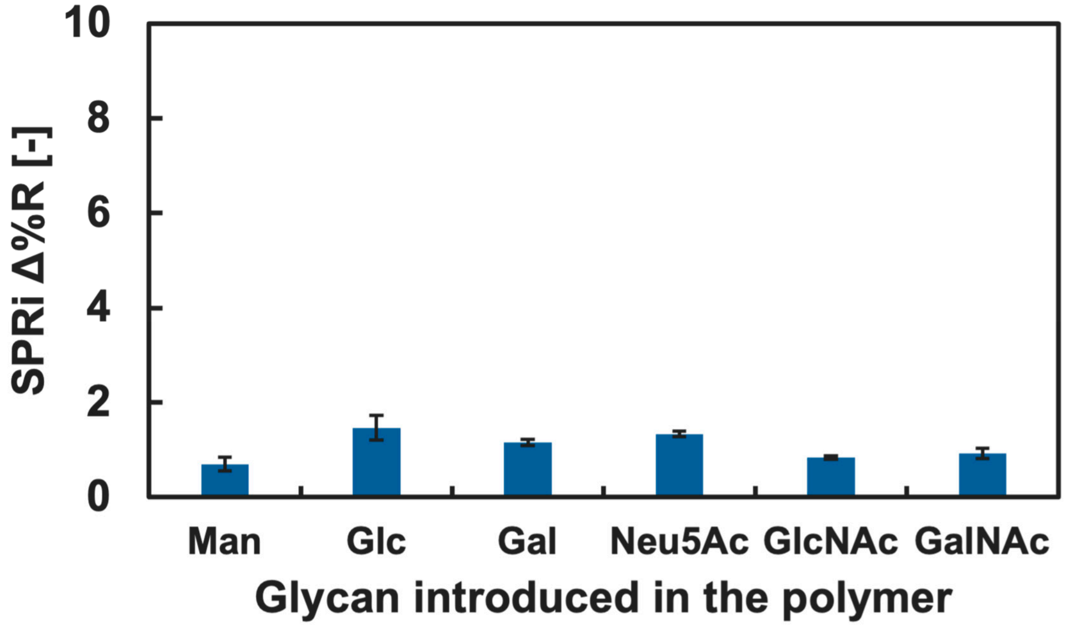

Figure S9. SPRi reflectivity changes obtained by addition of IL-1β onto the GM surfaces with different type of glycan.

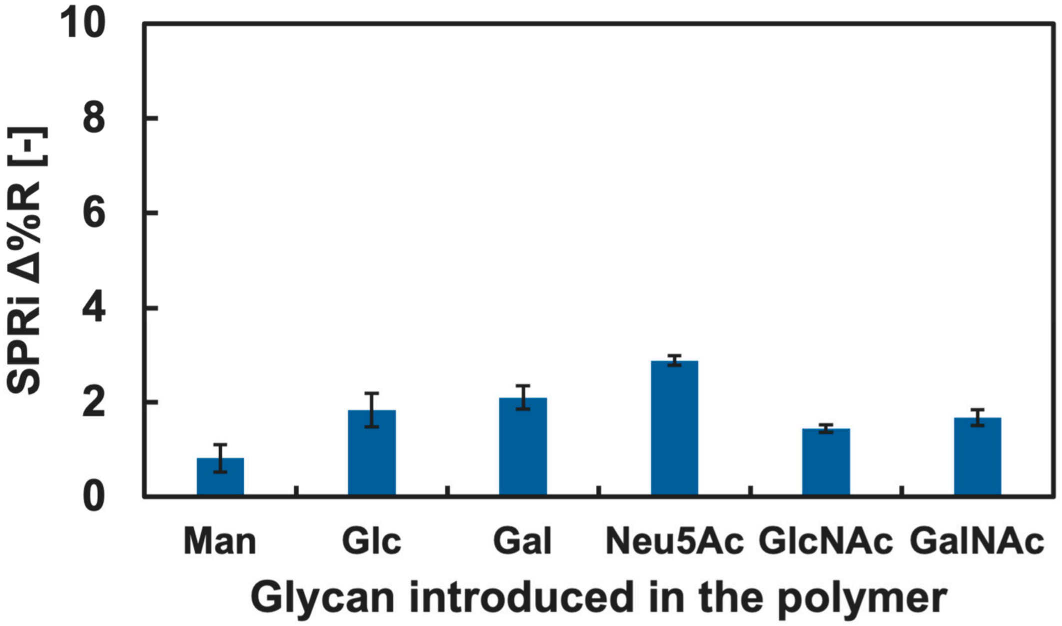

Figure S10. SPRi reflectivity changes obtained by addition of IL-6 onto the GM surfaces with different type of glycan.
